# Immunosuppressive cell populations as potential drivers of extrinsic immune checkpoint inhibition resistance in clear cell renal cell carcinoma

**DOI:** 10.3389/or.2026.1953850

**Published:** 2026-09-23

**Authors:** Usman Humayun Temuri

**Affiliations:** Loughborough College, Loughborough, United Kingdom

**Keywords:** clear cell renal cell carcinoma (ccRCC), immune checkpoint inhibitor resistance, immune checkpoint inhibitors (ICIs), tumor microenvironment, tumor-associated macrophages, regulatory T cells (Tregs), myeloid-derived suppressor cells (MDSCs), natural killer cells (NK cells)

## Abstract

Clear cell renal cell carcinoma (ccRCC) is an immunogenic cancer in which the use of Immune checkpoint inhibitors (ICIs) has provided clinical benefit. However, long-term therapeutic benefit is limited, as some do not respond initially while others develop resistance after treatment. Although extensive studies have been conducted on tumor intrinsic mechanisms, cells within the tumor microenvironment are also recognized as extrinsic factors in ICI resistance. A critical evaluation of the roles of tumor-associated macrophages, regulatory T cells, myeloid-derived suppressor cells, dysfunctional CD8^+^ T cells, natural killer cells, and dendritic cells in driving immune dysfunction in ccRCC has been provided in this narrative review. The evidence reviewed in this article suggests that these cell populations collectively impair antigen presentation, effector-cell activation, and cytotoxic function, while also promoting an immunosuppressive microenvironment. However, most of this evidence is collected from mechanistic or prognostic studies rather than from ICI-treated ccRCC cohorts. A single immune cell population cannot explain overall resistance to checkpoint blockade. Instead, resistance to ICIs due to extrinsic factors appears to result from multiple interactions among several immune cell populations. This emphasizes the need for studies that examine the function of immune cells in relation to ICIs to distinguish mechanisms that directly drive resistance to ICIs from general prognostic immune dysfunction.

## 1 Introduction

RCC accounts for approximately 2%–5% of all cancer malignancies, resulting in a higher occurrence rate in developed countries (1). Over 330,000 cases of RCC are diagnosed annually, with more than 140,000 deaths occurring worldwide due to RCC, ranking RCC amongst the 10 most commonly occurring cancers globally (1, 2). The most ubiquitous and most researched subtype of RCC is clear cell renal cell carcinoma (ccRCC); 75%–80% of all RCC cases are ccRCC (1). Several factors play a significant role in disease development, such as age, sex, smoking, hypertension, and obesity. Studies suggest that males are at higher risk of developing RCC as their age increases (1, 3). As one of the most common renal malignancies with a unique molecular landscape, ccRCC serves as a key model for investigating mechanisms of resistance to immune checkpoint inhibition.

Frequent molecular alterations such as the inactivation of the Von Hippel–Lindau (VHL) tumor suppressor gene located on chromosome 3p25 characterize ccRCC (4). It has been identified that the loss of VHL function, which occurs in a significant proportion of sporadic ccRCC cases, leads to the stabilization of hypoxia-inducible factors (HIF) under normal oxygen conditions (5, 6). Therefore, genes participating in angiogenesis, such as the vascular endothelial growth factor (VEGF), which assists in establishing a highly vascular environment of ccRCC, are overexpressed (7). Nevertheless, VHL-HIF-VEGF signaling not only supports angiogenesis and tumor growth but also helps establish conditions for immune evasion by remodeling the tumor microenvironment (8–10).

Cytokine-based therapies such as interleukin-2 (IL-2) and interferon-alpha (IFN-α) were formerly used to treat advanced RCC, but these therapies resulted in limited effectiveness in treating the disease (9). But, due to the passage of time, new therapies such as vascular endothelial growth factor (VEGF) receptor tyrosine kinase inhibitors and mTOR inhibitors have shown improvement in patient outcomes; however, these therapies also proved to be ineffective in some cases due to the development of resistance by the cancer (8).

Inhibitory signaling pathways that suppress T-cell activity are blocked by ICIs (immune checkpoint inhibitors), which are monoclonal antibodies, thereby providing anti-tumor immunity (11, 12). Programmed cell death protein-1 (PD-1) and cytotoxic T-lymphocyte-associated protein 4 (CTLA-4), which limit T-cell activation, are the most common targets in ccRCC (2, 13). Exploitation of these pathways by tumor cells via the upregulation of ligand PD-L1 leads to reduced proliferation of T cells, deteriorated cytokine production, and the development of T cell exhaustion (12, 13). While this paradigm has been successful in establishing checkpoint inhibition as a central ccRCC therapy, this model is based on the assumption that the removal of inhibitory immune signals is sufficient to reestablish an effective anti-tumor immune response. Hence, ICIs are used to inhibit these checkpoint interactions, which helps in reestablishing anti-tumor immune responses (11). Nevertheless, this simplified model is increasingly challenged by the variable clinical responses to ICIs in ccRCC, as not all immune-infiltrated tumors show a successful response to checkpoint blockade.

Despite their clinical benefits, a range of biological barriers limit the effectiveness of ICIs.

Amongst these factors lie the tumor-intrinsic factors such as genetic alterations and impaired antigen presentation, as well as extrinsic influences from the tumor microenvironment, including immunosuppressive cell populations, metabolic dysregulation, and the upregulation of alternative inhibitory pathways (14–16).

However, this review focuses specifically on immunosuppressive immune-cell populations as tumor-extrinsic contributors to ICI resistance. Since ICIs work by restoring anti-tumor immunity instead of killing tumor cells directly, immune cell populations that weaken presentation of antigens, suppress the activation of effector cells, cause exhaustion, reduce cytotoxicity, or shift inflammation towards the tumor support may also be plausible factors in establishing resistance to ICIs.

Moreover, this review does not treat poor prognosis as direct evidence of ICI resistance, because, while much of the evidence is based on ICI cohorts, much remains mechanistic or prognostic. But mechanisms that weaken antigen presentation, immune activation, or cytotoxic function remain relevant because ICIs depend on restoring these immune processes. This review therefore brings together mechanistic, prognostic, and emerging treatment-linked evidence and assesses how TAMs, Tregs, MDSCs, dysfunctional CD8^+^ T cells, NK cells, and DCs affect the ccRCC tumor microenvironment, support immune evasion, and may be contributors to resistance to immune checkpoint inhibition.

## 2 Molecular basis of immune remodeling in ccRCC

Although the main focus of this review is on immunosuppressive immune-cell populations as tumor-extrinsic contributors to ICI resistance, tumor-intrinsic alterations also play a significant role in shaping the landscape in which these processes arise. The loss of chromosome 3p and frequent inactivation of the von Hippel–Lindau (VHL) tumor suppressor gene are key driver events in the development of ccRCC, therefore stabilizing hypoxia-inducible factors (HIFs) and activating pro-tumorigenic pathways such as VEGF signaling (4, 5, 7). Such alterations play an important role in the development of an immunologically altered tumor microenvironment and the highly vascular nature of ccRCC (8–10, 17). However, the development of a tumor is not due to one single molecular pathway; instead, evidence across multiple studies suggests that a two-way relationship between hypoxia and genomic instability, alongside broader interactions between tumor genetics and the immune microenvironment, drives tumor development (18, 19).

Furthermore, while the core part of ccRCC biology is shaped by the VHL-HIF-VEGF axis, the ability of this axis to explain therapeutic resistance appears to be limited. Response to VEGF-targeted therapy or survival outcomes are not consistently predicted by the loss of VHL. Moreover, while the establishment of the immunosuppressive tumor microenvironment is promoted by VHL-driven angiogenesis, current evidence suggests that a single angiogenic pathway cannot be used to explain the resistance to ICIs in ccRCC (8, 9, 20, 21).

Altogether, these findings indicate that conditions favoring immune dysfunction are established by tumor-intrinsic factors; but these factors do not fully determine the therapeutic outcome. Therefore, understanding why immune cells fail despite successful recruitment requires a detailed examination of the tumor microenvironment, specifically the roles of tumor-associated macrophages, dysfunctional T cells, and other immunosuppressive cellular populations.

## 3 Immunosuppressive tumor microenvironment and immune dysfunction in ccRCC

### 3.1 Tumor-associated macrophages (TAMs)

As much as one-third of the immune cell population in some tumors consists of tumor-associated macrophages, highlighting the major role they play within the ccRCC tumor microenvironment (22). However, while frequent macrophage infiltration in RCC highlights their biological importance, their presence alone does not provide sufficient information about prognosis. Evidence from later ICI-treated cohorts suggests that, for distinguishing therapeutic responses, macrophage density may be insufficient. This therefore indicates that higher immune infiltration does not necessarily mean a higher degree of immune suppression (23, 24). Accordingly, the activation state of each TAM should be taken into account when understanding their function, as the number of TAMs is not as significant as their activation state, which is divided into the classically activated M1, and the alternatively activated M2.

#### 3.1.1 M1/M2 macrophages

Two macrophage states, classically activated M1 macrophages, which are associated with antigen presentation, pro-inflammatory responses, and tumoricidal activity, and M2-like macrophages, which support immunosuppression and tumor progression, exist across a functional spectrum within the tumor microenvironment (25, 26). It has been found that higher levels of M1-like macrophages have resulted in better patient outcomes and survival, whereas the infiltration of macrophages is largely dominated by M2-like phenotypes, especially in advanced stages of the disease (27–29). Correspondingly, higher levels of M2-like tumor-associated macrophages (TAMs) have consistently been associated with poorer clinical outcomes in RCC (30, 31). Nevertheless, confidence in the M1/M2 model should be interpreted cautiously, as much of this originates largely from *in vitro* models of macrophage polarization, where the macrophages often display mixed phenotypes simultaneously expressing pro- and anti-inflammatory markers (32). While the M1/M2 classification oversimplifies macrophage biology, understanding the mechanisms underlying the M1/M2 dichotomy as it pertains to the immunosuppressive function of TAMs in ccRCC is crucial.

#### 3.1.2 Mechanisms of TAM-driven immunosuppression

The role of TAMs in establishing an immunosuppressive tumor microenvironment is supported by several studies. The opposing contributions of the M1 and M2 macrophages to the tumor microenvironment are reflected by their differing functional properties. For example, M1 macrophages contribute to anti-tumor immunity by promoting phagocytosis and secreting pro-inflammatory cytokines, including TNF-α, IL-1β, IL-6, IL-12, and IL-23, thereby supporting both innate and adaptive immune responses (33–35). In contrast, factors such as IL-4, IL-10, IL-13, and CSF-1, as well as hypoxic and metabolically altered tumor conditions that favor fatty acid oxidation-dependent macrophage programming, promote M2 polarization (35–39). Production of high levels of IL-10, TGF-β, VEGF, and PD-L1 by M2-like TAMs promotes angiogenesis, immunosuppression, and tumor progression (40, 41). Daurkin et al. (42) demonstrated that greater quantities of IL-10 are secreted by larger tumors, suggesting that during tumor progression, macrophage-mediated immunosuppression intensifies. Monocytes recruited into the tumor tissue, along with myeloid-derived suppressors that infiltrate the tumor tissue and differentiate under the influence of cytokines and chemokines, are the primary precursors of TAMs (43, 44). Altogether, these studies provide enough evidence to support TAM-mediated immune regulation. However, this evidence is mostly derived from experimental or *ex vivo* studies, therefore limiting confidence in how directly these mechanisms influence clinical outcomes in patients. TAMs are also placed within a wider myeloid suppressive network.

An important clinical implication of these immunosuppressive mechanisms is the progressive loss of function of anti-tumor T cells, a hallmark of T-cell exhaustion that occurs in ccRCC.

#### 3.1.3 TAMs and T-cell exhaustion

In addition to their direct suppression of immune activity, TAMs also play a key role in driving overall immune dysfunction within the ccRCC tumor microenvironment. High levels of TAMs are associated with a suppressed immune landscape, characterized by reduced effector cytokine production, elevated Treg infiltration, NK-cell dysfunction, and increased expression of inhibitory immune checkpoints, including PD-1 and TIM-3 on T cells, collectively reflecting progressive T-cell exhaustion (22, 27–29). In accordance with these findings, Krishna et al. (45) has demonstrated that high TAM infiltration is also associated with reduced responsiveness to immune checkpoint inhibitors in RCC. However, evidence from other clinical studies has not consistently supported this claim. For example, Kazama et al. (24) identified that in metastatic RCC patients treated with ICIs, CD68^+^ macrophage infiltration has proved to be a favorable prognostic factor, which then contradicts the view that increased TAM infiltration necessarily predicts poorer immunotherapy outcomes. These findings suggest that there is insufficient evidence to establish a consistent clinical relationship, as TAM abundance alone cannot reliably determine ICI responsiveness. Given the high prevalence of exhausted T cells that express inhibitory immune checkpoints, the role of TAM-mediated checkpoint signalling in immunotherapy resistance has attracted a lot of interest.

#### 3.1.4 TAM-mediated checkpoint signaling as a driver of ICI resistance

Various studies that examined predictive biomarkers in RCC challenged the assumption that a major driver of ICI resistance is TAM-mediated checkpoint signaling. While some mechanistic models state that TAM-derived PD-L1 promotes immune evasion (40, 41), clinical evidence supporting this remains limited. For example, Raimondi et al. (23) stated that reliable predictive value for ICI response in metastatic RCC has not been demonstrated by any tumor microenvironment biomarker, including macrophage infiltration and PD-L1 expression. Similarly, it was reported by Labriola et al. (46) that no correlation was found between PD-L1 expression and response to treatment.

Altogether, these findings suggest that immunotherapy resistance cannot be explained by TAM-driven checkpoint upregulation alone, which suggests that treatment failure might be due to additional mechanisms. In recent years, however, research has broadened its focus from checkpoint regulation to signaling pathways that operate intracellularly and drive tumor growth and therapeutic resistance by TAMs.

#### 3.1.5 Signaling pathways linking TAMs to tumor progression

Recent studies show that multiple interconnected signaling pathways are used by TAMs to contribute to tumor progression. Through the activation of signaling pathways such as IL-6/STAT3, cytokines produced by TAMs, including IL-6, have been shown to promote epithelial–mesenchymal transition and metastatic behavior (47). Furthermore, additional evidence demonstrates that RCC invasion, migration, and tumor progression are facilitated by TAM-secreted chemokines such as CCL5 and CXCL13 (48, 49). RCC and TAM-derived exosomal lncRNAs and miRNAs have been shown to promote M2 polarization, angiogenesis, and tumor proliferation through STAT3 signaling and related mechanisms, suggesting that exosome-mediated communication also appears to reinforce this immunosuppressive network (50–52). Additionally, TAMs have been shown to activate AKT/mTOR signaling and TIM-3-related pathways, thereby reducing their responsiveness to targeted therapies and promoting stem-like characteristics in tumor cells (53, 54). Even though these studies provide us with substantial evidence on TAM involvement in tumor progression, the majority of these still rely on preclinical models.

Altogether, these findings indicate that, rather than merely passive components of the immune infiltrate, tumor-associated macrophages (TAMs) are active drivers of immune dysregulation in ccRCC. Although certain mechanistic models support TAM plasticity, some clinical data from studies determining the relationship between TAM infiltration and ICI responsiveness remains inconsistent and at times contradictory with respect to ICIs (24, 45). Furhtermore,loss of MHC-I antigen-presentation has been linked to progressive disease in an ICI-treated mRCC cohort (46). This suggests that it is unlikely that TAMs alone act as determinants of immunotherapy resistance, hence leaving their value as drivers of immunotherapy resistance unresolved.

Instead the current evidence places TAMs within a wider immunosuppressive network, Treg infiltration, dysfunction of NK-cells and T cell exhaustion have been linked to high TAM abundance (22, 27–29), while candidate interactions between TAM states and tissue-resident CD8^+^ T cells in an Ipi/Nivo-resistant patient have been identified by single-cell analyses (45). MRC1^+^FOLR2^+^ TAMs are further linked with terminal-effector Tregs by spatial and single-cell evidence, however this evidence is not directly linked to ICI response instead this evidence is mechanistic or computational (55). Therefore according to the current evidence TAMs are not established as direct mediators of ICI resistance, however this also does not support viewing them as markers of poor prognosis. Their functional contribution to an interconnected immunosuppressive microenvironment is supported by mechanistic evidence (22, 27–29, 45, 55). Whereas treatment-linked evidence remains limited and conflicting (24, 45, 46).

### 3.2 CD8^+^ T-cell dysfunction and exhaustion in ccRCC

CD8^+^ T cells contribute substantially to the detection and control of emerging malignancies, thus acting as key mediators in anti-tumor immune responses. They release perforin and granzymes that induce target cell death to eliminate cancer cells (56, 57). Nonetheless, in ccRCC, despite their high abundance, their ability to function and target tumor cells is frequently impaired, contributing to immune evasion and tumor progression, suggesting that, for successful anti-tumor activity, their presence alone is not enough.

#### 3.2.1 CD8^+^ infiltration and survival outcomes in ccRCC

The association between high CD8^+^ T-cell infiltration and poor survival outcomes is a notable paradox in ccRCC because, in most malignancies, high CD8^+^ T-cell infiltration is generally associated with a favorable prognosis. This is largely attributed to widespread T cell dysfunction rather than their absence, indicating that immune presence does not always translate into effective anti-tumor activity (22, 58). This has been interpreted as a consequence of T cell dysfunction rather than reduced immune infiltration. However, other studies do not support this interpretation. For example, Stenzel et al. (58) reported that in patients responding to ICI therapy, high cytotoxic T cell densities were found within the tumor, thus suggesting that an active anti-tumor response may be reflected by CD8^+^ T-cell infiltration. Furthermore, they also found that higher CTL density was associated with worse cancer-specific survival. Together, these findings suggest that the number of CD8^+^ T cells is not a reliable indicator of therapeutic response.

#### 3.2.2 IFN-γ signaling

Multiple mechanistic studies suggest that CD8^+^ T-cell dysfunction can be promoted by chronic immune activation. Antigen presentation is promoted by Interferon-γ (IFN-γ) through MHC-I, MHC-II, TAP1, and PSMB9; furthermore, Interferon-γ (IFN-γ) also induces inhibitory molecules including PD-L1, and B7-H3 (59–61, 63). This is accompanied by regulatory T cell expansion and dysfunctional CD8^+^ T cells, indicating coupled immune activation and suppression. NLRC5, B2M, and CIITA-mediated MHC-II expression are further increased by IFN-γ signaling via STAT1/IRF1, but the widespread checkpoint upregulation across epithelial, endothelial, and myeloid cells forms an immunosuppressive micro-environment that limits co-stimulation of the T cells (60, 61). IFN-γ serves as a bifunctional transcriptional regulator that supports immune activation, while also initiating a suppressive feedback pathway under chronic signaling conditions (62, 63). This dual role was then supported by Mojic et al. (64) who found that IFN-γ signaling plays a tumor-promoting role, potentially enabling immune evasion, suggesting that checkpoint induction may represent more than a simple negative feedback response. Together, these findings challenge the simplistic models in which checkpoint induction is viewed as a negative response, therefore suggesting the presence of a more complex relationship between immune activation and immune suppression.

#### 3.2.3 Stromal and metabolic contributors to T-cell exhaustion

T cell function is further impaired by chronic inflammatory and stromal mechanisms. Sustenance of myeloid-biased inflammation and the promotion of tolerogenic dendritic cells with reduced CCR7 and CD86 by NF-κB and STAT3 signaling limit effective preparation of T cells (65–67). This suggests that CD8^+^ T-cell dysfunction is not entirely cell-intrinsic, as TAMs, Tregs, MDSCs and dysfunctional DC states may converge on impaired T-cell activation and effector function, while several of the same suppressive pathways also impair NK-cell cytotoxicity (22, 27–29, 68–74). Lymphocyte invasion through extracellular matrix and vascular barriers is restricted by cancer-associated fibroblasts (CAFs) and endothelial remodeling (75). At the cellular level, CD8^+^ T cells undergo progressive exhaustion which is characterized by a decrease in perforin/granzyme expression, impaired mitochondrial biogenesis, and metabolic dysfunction caused by accumulation of lactate and lipid surplus (76). Terminal exhaustion, driven by mitochondrial ROS accumulation and suppression of PGC-1α signaling, is accelerated by chronic antigen exposure under hypoxia, linking metabolic stress to impaired immune responses and resistance to munotherapy (77). Furthermore, deprivation of glutamine has been shown to impair mitochondrial function and suppress perforin and granzyme B secretion in infiltrating CD8^+^ T cells, thereby intensifying T cell dysfunction (78). Similarly, increased PD-1 expression, decreased production of IFN-γ and TNF-α, and reduced mitochondrial membrane potential have been linked to increased CD38 expression on CD8^+^ T cells, further implicating mitochondrial dysfunction as a central driver of impaired anti-tumor immunity (79). Even so whether these pathways play a direct role or not remains unclear. Ma et al. (80) indicated that metabolic dysfunction is unlikely to be explained by a single pathway, as deficiency of CD38 was insufficient to restore the role of NAD^+^ levels or improve anti-tumor immunity. Although the source of this evidence is mostly experimental models rather than ccRCC-specific clinical cohorts, the assumption that individual metabolic regulators are sufficient to explain dysfunctional T-cells is challenged by this evidence, which suggests that exhaustion likely occurs due to several pathways that interact with each other rather than a single dominant mechanism.

While the exhaustion of T-cells is driven by increased expression of PD-L1 and other inhibitory receptors, Raghubar et al. (81) reported that exhausted and pro-tumor phenotypes may be displayed by the ccRCC immune microenvironment without increased expression of classical checkpoint molecules such as PD-1, PD-L1 or CTLA-4. Instead, others, including HAVCR2, LAG3 and TREM2, were identified. Hence, directly challenging models that link classical checkpoint overexpression to therapeutic resistance suggests that immune dysfunction may persist even in the absence of certain classical checkpoints.

Furthermore, the exhaustion of T-cell states is linked to transcriptional regulators such as TOX and their sustained expression, which are associated with features of dysfunction and chronic antigen stimulation (82–84). However, subsequent studies provide evidence of functional impairment in TOX-deficient T cells and of TOX expression in non-exhausted T-cell populations, thereby suggesting that a single transcriptional program cannot define exhaustion alone (85, 86). These findings suggest that CD8^+^ dysfunction results from several interacting mechanisms rather than a single pathway.

Overall, an important contributor to immune evasion in ccRCC has been identified as CD8^+^ T-cell dysfunction. According to multiple mechanistic studies, chronic inflammatory signaling, checkpoint induction, stromal remodeling, and metabolic dysfunction promote exhausted T-cell states. However, some clinical evidence from studies reports contradictory associations between CD8^+^ T-cell infiltration and patient outcomes (22, 58). Consequently, while CD8^+^ T-cell exhaustion remains a central feature of ccRCC biology, the mechanisms driving dysfunction and resistance to immunotherapy are likely more heterogeneous than current models suggest.

Therefore, CD8^+^ dysfunction may be caused not only by T-cell-intrinsic exhaustion pathways but also by the interactions which surround the suppressive immune cell populations. Immune environments rich with TAMs are linked to reduced production of effector cytokine and exhaustion of T cells (22, 27–29), while the suppression of effector T-cells and antigen presenting cells is carried out by Tregs (68, 69), MDSCs inhibit cell activity (70, 71), and effective T-cell priming may be impaired by tolerogenic DC (65–67). Some of these suppressive mechanisms also affect NK cells; for example, the cytotoxic activity of both CD8^+^ T cells and NK cells has been shown to be inhibited by Tregs (87).

This suggests that the dysfunction of CD8^+^ T cells reflects a coordinated cytotoxic immune activity rather than a defect occurring in isolation. Collectively, an interconnected model of CD8^+^ dysfunction is supported by these findings, although direct evidence establishing which of these cellular interactions independently drive ICI resistance remains limited.

Thus the dysfunction of CD8^+^ T cells has been supported by current evidence as a renowned contributor to impaired anti-tumor immunity, but does not establish CD8^+^ as an independent mediator of ICI resistance. Evidence from ICI-treated patients indicates that cytotoxic T-cell states differ between responders and non-responders (45, 58). Whereas inconsistent associations between CD8^+^ infiltration and clinical outcome indicate that cell abundance alone is primarily prognostic and cannot reliably predict ICI resistance (22, 58).

### 3.3 Regulatory T-cells (Tregs)

Secretion of IL-10 and TGF-β by Regulatory T cells (Tregs) inhibits effector T cells and antigen-presenting cells, leading to immunosuppression. Tregs are commonly characterized by high CD25 and FOXP3 expression (68, 69). Even though the infiltration of Tregs in ccRCC as compared to other tumors is low, it has been reported across several studies that there is an increased intratumoral Treg abundance in advanced disease, which is then associated with poorer clinical outcomes (88, 89). Nevertheless, an inconsistency has been found across studies in this relationship.

Siddiqui et al. (90) reported that CD4^+^CD25+Foxp3− T cells, which might play a role in shaping outcomes, were associated with death in RCC. Li et al. (91) reported that as the tumor stage increases, there is a reduction in circulating Treg populations, which suggests that the accumulation of Tregs in ccRCC might be due to redistribution into the tumor microenvironment rather than a uniform expansion of the overall Treg population. Therefore, while these findings highlight the importance of distinguishing local tumor infiltration from systemic immune changes, they also underscore the role of Tregs in immunosuppression.

#### 3.3.1 A possible relationship between treg infiltration and CD8^+^ T-cell exhaustion

T-cell suppression, inhibition of antigen-presenting cell function, and promotion of an immune environment that favors tumor progression have been linked to the immunosuppressive effects of Tregs (68, 69). In line with this, findings demonstrate that an increase in Treg infiltration commonly occurs alongside CD8^+^ T-cell exhaustion (45, 92). Which suggests that instead of just preventing immune cells from being recruited, Tregs may also contribute to maintaining the dysfunctional states of T-cells. This complements the earlier observation that rather than a reduction in immune infiltration, a major feature of ccRCC is CD8^+^ T cell dysfunction. Although high emphasis is put upon cytokine-mediated suppression by IL-10 and TGF-β (93), more recent evidence from Paluskievicz et al. (94), suggests that cytokine-mediated suppression represents only a small component of a much larger suppressive network and that multiple other mechanisms are interacting together to build an immunosuppressive environment. Consistent with this, expansion of Tregs can be promoted by MDSCs (70, 71), while spatial and single-cell analyses have linked terminal-effector Tregs with MRC1^+^FOLR2^+^ TAMs (55). The cytotoxic activity of both CD8^+^ T cells and NK cells can be suppressed by Tregs (87). Consequently, much like the oversimplified M1/M2 framework which was discussed previously, reducing Treg activity to cytokine production will probably oversimplify their role in immune dysfunction.

#### 3.3.2 PD-1 expression on Tregs

Studies report experiments suggesting that, compared to PD-1 counterparts, PD-1^+^ Tregs possessed less suppressive ability (95, 96). But, evidence from other studies challenges this interpretation. Tay et al. 2020 (97) proposed that resistance against PD-1-directed therapies may be due to Tregs expressing PD-1, while another study reported that after treatment with nivolumab, instead of the total Treg abundance, PD-1^+^ Tregs were associated with poorer response and survival outcomes (98). These findings suggest that instead of the abundance of Tregs, the functional phenotype of Tregs may be more relevant to ICI responses, while comparing evidence from both TAM and CD8^+^ T cell studies in which the abundance of cells proved to be an unreliable predictor of therapeutic response. Furthermore, evidence from another study supports the above information by indicating an incomplete understanding of interactions between the PD-1/PD-L1 axis and Treg biology, which may influence both Treg function and responses to checkpoint inhibition (99).

Overall, according to current evidence, Tregs play a vital role in building the immunosuppressive microenvironment of ccRCC. Nevertheless, conflicting findings regarding PD-1^+^ Treg function, alongside inconsistencies between tumor and peripheral studies, suggest that ICI resistance cannot be explained by the abundance of Tregs or by any single suppressive mechanism. Therefore, just like CD8^+^ T-cells and TAMs, Tregs also seem to function as part of a larger immunosuppressive network.

This network appears to be bidirectional, as suppressive myeloid populations might reinforce Treg activity, while Tregs themselves impair cytotoxic and antigen presenting immune functions. However, although after treatment with nivolumab (98), the evidence has associated PD-1^+^ Tregs with poorer outcome, most of this evidence on Treg crosstalk remains mechanistic and does not directly establish that these interactions cause ICI resistance.

Overall, prognostic studies, mechanistic findings and observations linked to treatment response all provide evidence on Tregs. Prognostic links often support higher levels of intratumoral Tregs (88–90).

Whereas more direct evidence of an association with treatment outcome is provided by poor response and survival following treatment with nivolumab in patients with PD-1+ Tregs (98). However, this does not establish Tregs as independent causal mediators of ICI resistance, particularly given conflicting evidence regarding the suppressive function of PD-1^+^ Tregs (95–99).

### 3.4 Myeloid-derived suppressor cells (MDSCs)

Across multiple studies, MDSCs have been consistently implicated in ccRCC-associated immunosuppression; rather than functioning in isolation, they link several immunosuppressive cell populations through inhibition of T-cell and NK-cell activity, promotion of Treg expansion, and their differentiation into M2-like TAMs (70, 71). In line with this, patients with ccRCC have elevated levels of circulating and intratumoral MDSCs. PMN-MDSCs, the most dominant subset of MDSCs in both tumor tissue and circulation, have been linked to increased expression of IL-8, CXCL5, and CCL3, as well as worse survival outcomes (100–102). Supporting this, a myeloid-associated gene signature, defined by IL-6, prostaglandins, and CXCL8-family chemokines, has been linked to poor patient outcomes with atezolizumab monotherapy (103). Together, these findings suggest that instead of MDSC presence itself, wider inflammatory programs alongside MDSC accumulation may be reflected by the adverse outcomes associated with MDSCs.

#### 3.4.1 Proposed suppressive mechanisms

Several overlapping pathways through which MDSCs suppress anti-tumor immunity include the production of arginase-1, TGF-β, IL-10, nitric oxide, and reactive oxygen species, as well as amino acid depletion mediated by arginase-1 and IDO1 (71, 104–106). Nonetheless, Sosnowska et al. (107) found that inhibition of arginase alone was insufficient to produce meaningful anti-tumor responses, suggesting that, despite arginase-mediated suppression’s contribution to immune suppression, MDSC dysfunction cannot be attributed to it alone. Despite MDSCs being most commonly studied in the context of the local tumor environment, Ku et al. (108) demonstrated that adaptive immunity can be impaired by circulating MDSCs at distant lymphoid sites through L-selectin downregulation. Even though these studies were not performed in ccRCC, the findings suggest that MDSC-mediated suppression is not confined to the tumor environment and may extend beyond it, further challenging single-mechanism models of immune dysfunction.

#### 3.4.2 A possible dual role in immune suppression and tumor-supportive remodeling

Alongside immune suppression, MDSCs have been associated with tumor progression through angiogenesis, invasion and metastatic dissemination via VEGF, BV8, FGF2, MMP9 and S100-A8/A9, while the recruitment of MDSCs alongside TAMs and Tregs is promoted by IL-1β-driven inflammation (109–112). These findings suggest that MDSCs might simultaneously reinforce immune suppression and tumor-supportive stromal remodeling. However, studies have reported a decrease in MDSC numbers when treated with anti-VEGF therapy (100). Sharma et al. (113) highlighted the restoration of alternative angiogenic pathways during treatment resistance, suggesting that, to overcome MDSC-associated tumor support, disrupting VEGF-dependent mechanisms alone may not be sufficient. Mirroring observations from TAMs and Tregs, where targeting individual pathways has not consistently translated into predictable clinical benefit.

#### 3.4.3 Uncertainty around MDSC subset classification and clinical significance

Although it has been proposed in early studies that M-MDSCs and PMN-MDSCs have distinct immunosuppressive and pro-angiogenic roles (114), later evidence reported that both populations can produce angiogenic and immunosuppressive mediators within the tumor microenvironment (112, 115), which challenges their functional classification. Moreover, biomarker development and functional characterization have been limited due to the absence of universally accepted phenotypic markers (110). Supporting this, Pawelec et al. (116) questioned whether MDSCs are exclusively pathological populations and noted that attempts to therapeutically reverse their effects have produced disappointing results. Consequently, while current evidence reports MDSCs as immune suppressive and tumor-promoting cell populations, it also suggests that MDSCs' clinical and biological significance is way more complex than what current models imply, and it is unlikely that MDSCs work as a singular targetable source for immune evasion.

Rather than acting through a single pathway these effects appear to form part of a broader immunosuppressive network, in which the activity of T- and NK-cell is impaired, Tregs expand, and macrophages are shifted towards an M2-like phenotype.

Thus the evidence supporting MDSCs in ccRCC is strongest at the mechanistic and prognostic levels. A biologically plausible role in immune dysfunction is played by their suppressive effects on T cells and NK cells, promotion of Treg expansion and M2-like macrophage differentiation, and association with adverse outcomes (70, 71, 100, 101, 102). Although a myeloid-associated gene signature has been linked to poorer outcomes with atezolizumab monotherapy (103), this does not establish MDSCs themselves as independent causal mediators of ICI resistance.

### 3.5 Natural killer (NK) cells

Natural killer (NK) cells are cytotoxic lymphocytes that play a role in eliminating tumor cells while also regulating immune responses through activating and inhibitory receptor signaling and cytokine secretion (117). The abundance of NK cells and their phenotype have been linked to disease stage and functional anti tumor capacity in RCC (118–120). However, findings from a clinical cohort involving IL-2 stimulation suggest that intratumoral CD56^+^ NK-cell infiltration alone was not significantly linked with objective response (121). Altogether, these findings suggest that the abundance of NK cells provides limited information about anti-tumor immunity. Studies that examine the function of NK cells within the tumor microenvironment support this interpretation. The balance between activating and inhibitory signaling has been associated with the activity of NK cells (117). Despite their high abundance within tumors, a reduced expression of multiple activating receptors has been exhibited in ccRCC by tumor-infiltrating NK cells (122). This suggests that cytotoxic function is not necessarily preserved by immune infiltration alone. Similarly, evidence from Thompson et al. (123), which was derived from murine models suggested that instead of sustained activation, persistent NKG2D engagement caused broad NK-cell desensitization. NK-cell function may be impaired by chronic receptor stimulation.

#### 3.5.1 A broader immunosuppressive network potentially contributing to NK-cell impairment

Rather than receptor signaling alone, a wider immunosuppressive network appears to drive NK-cell dysfunction. Tregs suppress NK-cell activity through TGF-β while limiting dendritic-cell-mediated IL-15 signaling, whereas MDSCs and TAM-associated adenosine signaling further impair NK-cell activation (72–74, 87, 124, 125). However, dendritic cells can exert opposing effects on NK cells by suppressing their function through IL-6 and IL-10 (126). Therefore, current evidence suggests that instead of being driven by a single suppressive pathway, NK-cell dysfunction arises due to interactions between multiple immunosuppressive cell populations.

Because several of these pathways also impair CD8^+^ T cell activity (72, 87), rather than as a single immune defect the dysfunction of NK-cells may occur alongside a broader loss of cytotoxic effector function.

#### 3.5.2 Variability in NK-cell dysfunction

Nevertheless, the dysfunction of NK-cells appears to be neither uniform nor irreversible across tumors. Trotta et al. (127) reported an increase in NK-cell activation and cytotoxicity in VHL-mutant ccRCC, which suggests that the function of NK-cells may be influenced by the genotype of the tumor. Similarly, although TGF-β is considered to be immunosuppressive. Foltz et al. (128) demonstrated that, while exhibiting reduced granzyme and perforin expression, activated NK-cells could become resistant to TGF-β-mediated cytokine suppression, which indicated that cytotoxicity is not necessarily restored by preserved inflammatory signaling. Likewise, despite the evidence that NK-cell activation is enhanced by IL-2. Han et al. (129) found that this alone failed to significantly inhibit RCC growth *in vivo*. Altogether, these findings suggest that to overcome the broader immunosuppressive tumor microenvironment, restoration of NK cells may be insufficient.

Overall, current evidence supports the important role NK cells play in anti-tumor immunity in ccRCC. Nevertheless, preserved infiltration, receptor activation, and cytokine responsiveness do not consistently translate into effective tumor control. Collectively, interactions between NK cells and other cell populations, such as Tregs, MDSCs, TAMs, and dendritic cells, reflect the broader immunosuppressive tumor microenvironment rather than isolated defects. Evidence states that rather than acting as a direct mediator of ICI resistance NK cell dysfunction is a mechanistic and prognostic feature of impaired anti-tumor immunity. In CheckMate-025 a dysfunctional NK-cell transcriptional signature was associated with worse survival, however it was not associated with progression-free survival or radiographic response to nivolumab or everolimus (130), indicating that its prognostic relevance is currently stronger than its predictive value for ICI resistance.

### 3.6 Dendritic cells (DC)

Dendritic cells (DCs) play a vital role in achieving anti-tumor immunity, as they can present tumor antigens, provide co-stimulatory signals, and promote T-cell differentiation, thereby linking innate and adaptive immune responses (131, 132). But evidence suggests that their presence defines their function in ccRCC less than their functional state and spatial arrangement do. Consistent with this, studies have found that ccRCC tends to contain more immature non-tertiary lymphoid structure (TLS)-associated DCs than mature ones. These immature DCs are linked to greater Treg and macrophage infiltration, a more immunosuppressive tumor microenvironment, and poorer clinical outcomes (92, 133, 134). Moreover, higher levels of mature TLS-associated dendritic cells are linked to better prognosis. This suggests that effective T-cell responses may depend not only on the number of lymphocytes present, but also on whether antigen presentation is functionally competent (134). Collectively, these findings suggest that despite substantial immune infiltration, impaired DC function may contribute to ineffective anti-tumor immunity. Therefore the dysfunction of DCs may extend beyond defective antigen presentation, as T cell priming can be weakened by impaired DC-mediated signaling, while altered DC–NK-cell communication may further reduce cytotoxic immunity (124–126, 131, 132).

#### 3.6.1 Possible mechanisms behind DC dysfunction

Nevertheless, rather than a simple mature-versus-immature distinction, the mechanism driving DC dysfunction appears more complex. Immature DCs may play an active role in suppressing immunity by promoting immunosuppressive T-cell populations via TGF-β and IL-10 signaling, even though DCs are often considered ineffective at antigen presentation (135). Similarly, while it has been proposed that a reduction in mature DCs is linked to impaired antigen presentation and the promotion of ineffective T-cell infiltration in ccRCC, Ataera et al. (136) reported that despite Treg depletion, tumor-associated defects in antigen presentation persisted, suggesting that, rather than secondary immunosuppression alone, DC dysfunction may also arise through tumor-intrinsic conditioning.

Although these studies were not performed in ccRCC, they collectively suggest that defective antigen presentation arises from multiple interacting mechanisms rather than from DC immaturity alone.

#### 3.6.2 Uncertain clinical significance

The clinical importance of DCs is difficult to assess, as they can vary widely in function. Although favorable outcomes may be due to mature TLS-associated DCs. Zhao et al. (137), showed that TLSs themselves may still contain immunosuppressive populations, including Tregs and dysfunctional B cells. Therefore, the presence of TLS alone cannot be taken as evidence of effective anti-tumor immunity. Similarly, broader evidence suggests that rather than fitting into fixed mature or immature categories, tumor-infiltrating DCs exist across a wide spectrum of functional states. In response to tumor-derived signals, tolerogenic features can be acquired by both mature and immature DC populations (138, 139).

Consequently, although DC function is consistently associated with immunosuppression and poorer prognosis in ccRCC, its relationship to ICI response remains unclear. Therefore, impaired DC function may contribute to ineffective checkpoint blockade, although it remains unclear whether ICI resistance is caused directly by specific DC phenotypes.

### 3.7 High-resolution single-cell perspectives on immune heterogeneity and cellular crosstalk

Computational clustering and differential gene-expression analyses may be used alongside High-resolution single-cell approaches. These High-resolution single-cell approaches may aid in overcoming the limitations of broad immune cell classifications by revealing functional heterogeneity. Krishna et al. identified 5 CD8^+^ T-cell clusters and 4 TAM clusters in multiregional ccRCC (45). Marked TAM enrichment and T-cell exclusion were observed by an Ipi/Nivo-resistant patient. In contrast, a higher number of tissue resident CD8^+^ T cells and low infiltration of TAM was reported in a complete responder. In the resistant patient CellPhoneDB analysis suggested potential suppressive interactions between HLA^hi/int TAMs and tissue-resident CD8^+^ T cells (45).

However, only six patients were included in this treatment linked study and out of these six only four were ICB-treated (immune checkpoint blockade). Furthermore, functional studies in larger patient groups are needed to confirm these cellular associations (45). This level of detail was extended by Long et al. by combining scRNA-seq with scATAC-seq,distinct transcriptional and epigenomic CD8^+^ T-cell states were identified and it was demonstrated that amongst all immune cell populations, the highest number of ligand-receptor interactions were formed by Tams. Interactions involving TAMs, Tregs, and proliferating or exhausted CD8^+^ T-cell populations were also identified using CellPhoneDB (140). In CheckMate-025 a transcriptional signature which was derived from exhausted CD8^+^ T cells was linked to poor survival; this reflects an association with treatment outcome rather than direct evidence that the identified state itself causes ICI resistance (140). Importantly this cellular heterogeneity is not limited to TAMs and CD8^+^ T cells. Six distinct Treg states, four macrophage subsets, and both conventional and plasmacytoid dendritic-cell populations were identified by Song et al. (55). Terminal-effector Tregs were linked with MRC1^+^FOLR2^+^ TAMs by spatial analyses and CellPhoneDB, while functional experiments showed that CD8^+^ T-cell proliferation and IFN-γ production were more strongly suppressed by IL-1β^+^ terminal-effector Tregs than conventional Tregs (55). For coordinated immunosuppressive activity, these findings provide mechanistic and spatial evidence. However, they do not provide direct evidence for ICI resistance; the authors also establish that additional cellular interaction could remain unresolved due to small sample size and limited validation experiments (55). In primary and bone-metastatic ccRCC single cell profiling has also resolved four MDSC and ten macrophage subpopulations, While possible interactions between macrophage subtypes and inhibitory T-cell populations were identified by CellPhoneDB (141). However, only three patients with bone metastases who received both a TKI and PD-1 inhibitor were included in the treatment comparison thus making it difficult to attribute the observed immune differences specifically to checkpoint blockade (141). Similarly Xu et al. identified six NK-cell subsets including a bright-like state which was enriched in advanced ccRCC and associated with reduced expression of cytotoxicity genes (130). A reduction in degranulation and cytotoxic activity in tumor-derived CD49a^+^CD9^+^ NK cells was demonstrated by functional experiments (130). Although, in Checkmate-025 this transcriptional signature was linked to worse survival outcomes, no association was shown by this signature with progression-free survival or radiographic response to nivolumab or everolimus, suggesting that it is mainly prognostic rather than clearly predictive (130).

Altogether, these findings indicate that broad immune-cell categories can be refined into distinct functional states by single-cell, multiomic and spatial approaches. These approaches can also identify candidate communication networks consistent with an interconnected model of immune dysfunction in ccRCC, by using ligand receptor analyses. Nevertheless, across these population treatment-linked evidence remains uneven and the conclusion that these states or interactions are the independent drivers of ICI resistance in ccRCC cannot be established by either transcriptional subclustering or computationally inferred cell–cell interactions alone.

## 4 Integrated synthesis of findings and future perspectives

### 4.1 Integrated synthesis of findings

Collectively, the reviewed evidence suggests that a single dominant suppressive population is unlikely to cause extrinsic immune resistance in ccRCC alone. Instead, ccRCC appears to have a functionally inefficient tumor immune microenvironment despite having high immune infiltration, as the presence of immune cells alone does not necessarily cause coordinated anti-tumor immunity. This pattern is evident across multiple immune cell populations in ccRCC: CD8^+^ T cells in high abundance may not necessarily result in anti-tumor immunity, as they might be dysfunctional or exhausted; high infiltration by NK cells may not prove to be effective either, as they might show reduced activating receptor expression, and DCs may show functionally impaired antigen-presentation and T-cell priming (22, 76, 92, 122, 133, 134). Therefore, the main issue in ccRCC is whether immune cells remain capable of supporting cytotoxic killing and immune activation.

Together, these findings suggest that suppression of immune response in ccRCC converges on impaired effector immunity, especially the dysfunction of CD8^+^ T-cells. Both effector T-cells and antigen-presenting cells can be suppressed by Tregs. The exhaustion of T cells and the reduced production of effector cytokines are associated with high TAM abundance; in addition, MDSCs weaken the activity of T cells and NK cells while promoting the expansion of Tregs and the differentiation of M2-like macrophages (22, 27, 68–71, 93). Suppression by other immune cells, including Tregs that produce TGF-β, reduced DC-mediated IL-15 signaling, and suppressive pathways linked to myeloid cells, further reinforce NK cell dysfunction (72–74, 87, 124, 125). These interactions suggest that overlapping suppressive pathways that weaken the presentation of antigens, activation of effectors, and cytotoxicity drive immune dysfunction in ccRCC.

This integrated model may give an explanation for why inconsistent prognostic and predictive significance is shown by individual immune cell populations. For example, both poor survival and improved ICI responses have been linked to high CD8^+^ T-cell infiltration [22, 58]. While, on the other hand, high infiltration of macrophages has been linked to reduced ICI response in some studies (45), and improved outcomes in others (24). A similar sort of uncertainty is also evident in Tregs.

Even after treatment with nivolumab, PD-1^+^ Tregs were linked to poorer outcomes, although both their ability to suppress and their contribution to resistance against PD-1-directed therapy remains uncertain (95–98), and in the biology of NK cells, where their infiltration into the tumor microenvironment appears less informative than their activation state (121). A similar pattern is shown by DCs too; mature DCs who are found within TLSs are commonly linked to better prognosis; however, suppressive immune cell populations may be present in TLS (92, 135, 138).

Therefore, the contradictions in literature are unlikely to reflect methodological differences across studies alone. They may also reflect a feature of ccRCC, in which effective anti-tumor activity may be indicated by immune cell infiltration. Still, in some other cases they may represent exhausted or suppressive inflammation.

Importantly, poor prognosis should not be concluded as direct proof of ICI resistance, as much of the evidence is provided by mechanistic or prognostic studies rather than ccRCC patients treated with ICIs. Nevertheless, this evidence is not uniform in strength; evidence directly linked to treatment is now more relevant than studies on infiltration alone. For example, strong evidence for a possible role of PD-1^+^ Tregs in resistance to ICIs can be their association with poor response and survival after nivolumab treatment (98).

This argument is further supported by interventional findings: *in vivo*, IL-2-enhanced activation of NK cells failed to significantly stop RCC growth (129). MDSC numbers were reduced by anti-VEGF therapy (100), and the inhibition of arginase was not enough to produce a significant anti-tumor response (107). Similarly, while still showing reduced expression of granzyme and perforin, activated NK cells may become resistant to TGF-β-mediated cytokine suppression, which indicates that cytotoxic ability is not necessarily restored by the restoration of one aspect of the immune function (128).

Nevertheless, these findings do not show that ICI resistance is caused by any single immune cell population. Instead, they suggest that despite the efforts made to recover anti-tumor immunity, harmful immune states may be more important clinically when they are present.

Overall, rather than the recruitment of immune cells alone, extrinsic resistance is best understood as the failure of coordinated immune function between immune cells. Conditions that are favorable to immune dysfunction might be established by Tumor-intrinsic alterations including VHL loss, HIF stabilization, and VEGF signaling (4, 5, 7–9).

However, the way that suppressive immune cell populations interact with each other is what the ongoing immune escape depends upon. Defective immune priming, suppressive myeloid inflammation, Treg-mediated inhibition, NK-cell dysfunction, or established CD8^+^ T-cell exhaustion may not be fully reversed by checkpoint blockade, even though checkpoint blockade can reduce the signaling of inhibitory receptors. The key findings regarding these immunosuppressive cell populations are summarized in Table 1.

**TABLE 1:** Summary of immunosuppressive cell populations in the ccRCC tumor microenvironment.

Cell populations	Association with prognosis	Proposed mechanism(s) of immune suppression	Evidence relevant to ICI response	Evidence types and limitations
Tumor associated macrophages (TAMs)	An increased number of suppressive/M2-like TAM states has been linked to poorer survival (27–31)	IL-10, TGF-β, VEGF, and PD-L1 are secreted by M2-like TAMs, thereby promoting angiogenesis and immune suppression, which is associated with reduced effector cytokine production, elevated treg infiltration, NK-cell dysfunction, and T-cell exhaustion (PD-1, TIM-3) (22, 27–29, 40–42)	In a small single-cell cohort enrichment of TAMs and the exclusion of T cells were observed in an Ipi/Nivo-resistant patient (45).In contrast to this in an ICI-treated RCC cohort the infiltration of CD68^+^ macrophages was linked to favorable outcomes (24). While macrophage-associated markers and PD-L1 have shown inconsistent predictive value (23, 46)	Mixed evidence: Mechanistic evidence for TAMs is largely experimental/*ex vivo* (40–44). (45) was a very small multiregional single-cell study, whereas (24) was a single center ICI-treated observational cohort Major limitation: An independent role of TAMs in ICI resistance is not established due to the presence of TAM heterogeneity, oversimplification by the M1/M2 framework and conflicting treatment-linked associations (24, 32, 45, 46)
CD8^+^ T cells	Poor survival in ccRCC is paradoxically associated with high infiltration of CD8^+^ T cells. However, amongst ICI responders a high cytotoxic T cell density has also been observed, indicating that clinical outcomes cannot be reliably defined by abundance alone (22, 58)	Alongside presentation of antigens, chronic IFN-γ signaling, metabolic stress, defective T cell priming, stromal restriction and transcriptional exhaustion programs can impair cytotoxic function (59–66, 76–86). CD8^+^ activity may be further impaired by suppressive myeloid populations and Tregs (68–74, 87)	In a complete ICI responder the enrichment of tissue resident CD8^+^ T cells was observed, where the enrichment of TAMs and exclusion of T cells were observed in a resistant tumor (45). Higher cytotoxic T-cell density was also reported among nivolumab responders (58). In CheckMate-025, an exhausted CD8^+^ transcriptional signature was associated with poorer survival (140)	Mixed human and experimental evidence: (58)included a large prognostic ccRCC series but only a very small nivolumab-treated subgroup; in a six-patient single cell study only 4 patients were treated with ICB (45) Major limitations: Infiltration, exhaustion phenotype and treatment response are not interchangeable and according to the current evidence dysfunctional CD8^+^ T cells states are not established as independent causal mediators of resistance (45, 58, 140)
Regulatory T cells (Tregs)	Increased intratumoral abundance in advanced disease is associated with poorer outcomes, although results differ between tumor and peripheral-blood studies (88–92)	T-cells and antigen presenting cells are suppressed via the secretion of IL-10/TGF-β and direct inhibition of cytotoxic lymphocytes by Tregs (68, 69, 87, 93, 94); spatial and single-cell evidence further links suppressive terminal-effector treg states with macrophage populations (55)	Samples from CheckMate-025 demonstrated that shorter PFS and OS were shown by patients receiving nivolumab, who had a higher proportion of intratumoral PD-1^+^ Tregs (98). However, experimental studies disagree on whether PD-1 expression increases or restrains treg suppressive function (95–99)	Relatively strong treatment-linked but correlative evidence: Specimens from the multicenter phase III CheckMate-025 trial were analyzed in (98). Major limitation: PD-1+ Tregs were associated with nivolumab outcome by the biomarker analysis however it did not experimentally establish that these cells caused resistance; PD-1^+^ treg biology also remains context-dependent (95–99)
Myeloid-derived suppressor cells (MDSCs)	A high number of circulating and intratumoral MDSCs have been associated with poor survival; high amounts of IL-8, CXCL5, and CCL3 have been linked to PMN-MDSCs (100–102)	MDSCs can suppress T and NK cell function, promote treg expansion and M2-like macrophage differentiation (70, 71).This suppression involves arginase-1, TGF-β, IL-10, nitric oxide, reactive oxygen species and amino-acid depletion while MDSCs can also promote angiogenesis and tumor-supportive remodeling (104–112, 115)	In the randomized phase II IMmotion150 study, reduced activity of atezolizumab monotherapy was linked to a myeloid-inflammatory transcriptional program (103)	Preclinical mechanisms plus large trial-derived correlative evidence: Evidence for many specific MDSCs mechanisms is largely based on experimental models (70, 104–108), whereas (103) was a large randomized clinical trial. Major limitation: The ICI-associated signature is not MDSC-specific, M-MDSC/PMN-MDSC phenotypes overlap, and direct causal evidence for MDSC-mediated ICI resistance remains insufficient (110, 114–116)
Natural killer cells (NK cells)	An increase in their infiltration has been linked to a favorable prognosis (118–120),whereas a dysfunctional bright-like NK-cell transcriptional state was associated with advanced disease and poorer overall survival (131)	Tumor-infiltrating NK cells may lose activating receptors and cytotoxic capacity (122,123). NK-cell activity may be further suppressed by Tregs, MDSCs, TAM-associated adenosine signalling and altered DC-derived signals (72–74, 87, 124–126)	In CheckMate-025 a dysfunctional bright-like NK-cell signature was associated with poorer overall survival however it was not associated with radiographic response or PFS under nivolumab or everolimus (130)	Single-cell/functional discovery plus external clinical validation: (130) analyzed scRNA-seq data from 13 patients, performed functional testing, and evaluated the derived signature in independent datasets including CheckMate-025 Major limitation: Rather than being clearly predictive of ICI response the clinical association is mainly prognostic, and direct NK mediated resistance has not been established (130)
Dendritic cells (DCs)	Immature/non-TLS-associated DCs are associated with greater infiltration of Tregs and macrophages and poorer outcomes, whereas mature TLS-associated DCs are linked to more favorable prognosis (92, 133, 134)	Effective T cell priming can be weakened by impaired antigen presentation and co-stimulation (131, 132). T-cell populations may be promoted by immature or tolerogenic DC states via TGF-β/IL-10 signaling, while tumor-derived signals can induce tolerogenic features across DC maturation states (65–67, 135–139)	Current evidence regarding DC dysfunction is predominantly mechanistic or prognostic (131–138, 142), among the studies reviewed here, specific DC phenotypes have not been directly validated as predictors of ICI response or resistance in ccRCC; therefore, direct treatment-linked evidence remains insufficient	Predominantly observational and mechanistic evidence: Both human prognostic studies and experimental research are included in the evidence, however rather than ccRCC itself some experimental findings come from other cancer types or preclinical models (92, 134–138) Major limitation: Mature/immature classification does not fully define DC functional heterogeneity, and specific DC states have not been validated as causal mediators or predictive biomarkers of ICI resistance

### 4.2 Major controversies and gaps

Whether immunosuppressive cell populations are the major drivers of ICI resistance or are markers of a dysfunctional tumor immune microenvironment is the central unresolved issue. According to some studies, infiltration of TAMs has been linked to reduced response to ICIs (45). But, the infiltration of CD68^+^ macrophages has been associated with favorable outcomes in ICI-treated metastatic RCC cohorts (24). Similarly, the infiltration of CD8^+^ T-cells has been linked to poor survival in RCC (58). In contrast, in patients who responded to ICI therapy, a higher density of cytotoxic T cells was reported (58). These contradictions suggest that without directly causing treatment failure, immune populations may be associated with clinical outcomes. It therefore remains unclear whether they cause resistance to ICIs and impair immune function from the outset, help maintain it over time, or simply accumulate within an already dysfunctional tumor microenvironment.

A related gap is that prognostic, mechanistic, and predictive evidence are often treated as if they provide the same type of conclusion. Higher levels of intra-tumoral Tregs have been linked to poor clinical outcomes (88–90). Nonetheless, this does not mean that Tregs are direct predictors of ICI failure. MDSC accumulation has been linked to poor survival outcomes and inflammatory myeloid programs (100–102). A myeloid-associated gene signature has been associated with poor outcomes after monotherapy with atezolizumab (103). Together, these findings show why poor prognosis should not be taken as direct evidence of ICI resistance and highlight the importance of suppressive immune states.

Another issue is that many immune cell groups are too broad to reflect their functional differences. Macrophage co-expression of inflammatory and suppressive markers limits M1/M2 macrophage function (32). Similarly, distinguishing between M-MDSCs and PMN-MDSCs is complicated as both release pro-angiogenic and immunosuppressive mediators suppress immune response and promote angiogenesis (112, 115). A similar problem can be seen in DCs, when DCs are exposed to tumor derived signals both mature and immature populations can become tolerogenic (138, 139), and in Tregs, where in some contexts the PD-1^+^ Tregs have been described as being less suppressive (95, 96), PD-1^+^ Tregs are associated with poorer responses following nivolumab use in ccRCC (98). From these examples, we can conclude that the states of immune cells and their functional context are more significant than abundance or single-marker expression.

Finally, a gap between detailed mechanistic evidence and evidence from clinical cohorts presents a limitation in the literature of the field. Several ways in which suppressive immune populations can weaken antigen presentation, effector-cell activation, and cytotoxic function, including MDSC-mediated suppression through arginase activity, TGF-β-associated NK-cell dysfunction, and tolerogenic DC programming, have been made evident by experimental and *ex-vivo* studies (107, 128, 138, 139). Although recent single-cell studies have allowed these functional states to be examined in greater detail clinical studies often rely on broad markers such as CD68, FOXP3, PD-1, PD-L1, or total immune-cell density; these markers do not distinguish successful anti-tumor immunity from dysfunctional or suppressive immune states (23, 24, 46).

### 4.3 Conclusion and future perspectives

Future studies should determine where anti-tumor immunity is being inhibited instead of treating CD8^+^ T cells, TAMs, Tregs, MDSCs, NK cells, or DCs as isolated biomarkers; this should be done by conducting further studies on immunosuppressive mechanisms such as defective priming, impaired cytotoxicity, suppressive myeloid inflammation, regulatory-cell activity, or loss of immune coordination as this framework may be more significant clinically than measuring immune cell abundance.

For the development of biomarkers, this particular shift is necessary; lineage markers alone cannot be used to reliably distinguish between protective immunity and dysfunctional inflammation, as inconsistent predictive value has been shown by broad markers such as CD68 and PD-L1 (23, 24, 46). Future biomarkers should therefore combine immune-cell identity with measures of cell function, such as cytotoxic capacity, antigen-presentation capacity, suppressive cytokine signaling, myeloid inflammation, and exhaustion states.

Therefore, in future studies longitudinal single-cell and spatial profiling of ICI-treated ccRCC should be combined with functional validation of candidate immune-cell states and ligand–receptor interactions which will allow to distinguish computationally inferred networks from interactions which influence treatment resistance directly. A useful approach would be to prospectively follow ccRCC patients while they receive ICI treatment, collecting paired tumor biopsies and blood samples before treatment, during the early stages of therapy. Overtime state changes in TAMs, Tregs, MDSCs, CD8^+^ T-cells, NK cells and DCs can be examined using single-cell transcriptomic and spatial analyses. These changes could be compared with clinically relevant outcomes such as treatment response, progression-free survival and radiographic progression. Experimental tests should be conducted on cellular states or ligand-receptor interactions linked to resistance, using approaches such as tumor–immune-cell co-culture or pathway perturbation. This would help determine whether they directly impair cytotoxic or antigen-presenting activity, rather than simply being associated with poorer outcomes.

Validation of these findings from initial cohorts should be done in independent groups of ICI-treated patients using predefined immune signatures. This would help determine whether functional immune states can predict treatment outcomes more effectively than broad markers such as CD68, FOXP3 or overall CD8^+^ T-cell density.

This review holds its significance by explaining how both immune infiltration and ICI resistance can occur alongside each other, without assuming that poor prognosis directly proves resistance to treatment. When defective priming, suppressive myeloid inflammation, Treg-mediated inhibition, impairment of NK cells, and established CD8^+^ T-cell exhaustion persist together, the effectiveness of ICIs may be reduced even though checkpoint blockade reduces inhibitory receptor signaling.

Hence, future studies should more directly examine the function of immune cells in ICI-treated cohorts. This would help in distinguishing between immune dysfunction that is linked to poor prognosis and immune dysfunction that genuinely predicts or contributes to immune checkpoint inhibitor resistance.

## Glossary

**AKT**: Protein kinase B
**B2M**: Beta-2-microglobulin
**B7**-**H3**: B7 homolog 3
**BV8**: Prokineticin-2
**CAFs**: Cancer-associated fibroblasts
**CCL3**: C-C motif chemokine ligand 3
**CCL5**: C-C motif chemokine ligand 5
**ccRCC**: Clear cell renal cell carcinoma
**CCR7**: C-C chemokine receptor type 7
**CD8**: Cluster of differentiation 8
**CD25**: Cluster of differentiation 25
**CD38**: Cluster of differentiation 38
**CD68**: Cluster of differentiation 68
**CD86**: Cluster of differentiation 86
**CIITA**: Class II major histocompatibility complex trans activator
**CSF-1**: Colony-stimulating factor 1
**CTL**: Cytotoxic T lymphocyte
**CTLA-4**: Cytotoxic T-lymphocyte-associated protein 4
**CXCL5**: C-X-C motif chemokine ligand 5
**CXCL8**: C-X-C motif chemokine ligand 8
**CXCL13**: C-X-C motif chemokine ligand 13
**DCs**: Dendritic cells
**EMT**: Epithelial–mesenchymal transition
**FGF2**: Fibroblast growth factor 2
**FOXP3**: Fork head box P3
**HAVCR2**: Hepatitis A virus cellular receptor 2
**HIF**: Hypoxia-inducible factor
**ICIs**: Immune checkpoint inhibitors
**IDO1**: Indoleamine 2,3-dioxygenase 1
**IFN**-α: Interferon-alpha
**IFN-γ**: Interferon-gamma
**IL-1β**: Interleukin-1 beta
**IL-2**: Interleukin-2
**IL-4**: Interleukin-4
**IL-6**: Interleukin-6
**IL-8**: Interleukin-8
**IL-10**: Interleukin-10
**IL-12**: Interleukin-12
**IL-13**: Interleukin-13
**IL-15**: Interleukin-15
**IL-23**: Interleukin-23
**IRF1**: Interferon regulatory factor 1
**LAG3**: Lymphocyte activation gene 3
**MDSCs**: Myeloid-derived suppressor cells
**MHC-I**: Major histocompatibility complex class I
**MHC-II**: Major histocompatibility complex class II
**M-MDSCs**: Monocytic myeloid-derived suppressor cells
**MMP9**: Matrix metalloproteinase 9
**mTOR**: Mechanistic target of rapamycin
**NAD+**: Nicotinamide adenine dinucleotide
**NF-κB**: Nuclear factor kappa B
**NK cells**: Natural killer cells
**NLRC5**: NOD-like receptor family CARD domain-containing protein 5
**NKG2D**: Natural killer group 2 member D
**PD-1**: Programmed cell death protein 1
**PD-L1**: Programmed death-ligand 1
**PGC-1α**: Peroxisome proliferator-activated receptor gamma coactivator 1-alpha
**PMN-MDSCs**: Polymorphonuclear myeloid-derived suppressor cells
**PSMB9**: Proteasome subunit beta type 9
**RCC**: Renal cell carcinoma
**ROS**: Reactive oxygen species
**S100-A8/A9**: S100 calcium-binding proteins A8/A9
**STAT1**: Signal transducer and activator of transcription 1
**STAT3**: Signal transducer and activator of transcription 3
**TAMs**: Tumour-associated macrophages
**TAP1**: Transporter associated with antigen processing 1
**TGF-β**: Transforming growth factor beta
**TIM-3**: T-cell immunoglobulin and mucin-domain-containing protein 3
**TLS**: Tertiary lymphoid structures
**TME**: Tumour microenvironment
**TNF-α**: Tumour necrosis factor alpha
**TOX**: Thymocyte selection-associated high mobility group box protein
Tregs: Regulatory T cells
**VEGF**: Vascular endothelial growth factor
**VHL**: Von Hippel–Lindau
**VISTA**: V-domain Ig suppressor of T-cell activation
**scRNA-seq**: single-cell RNA sequencing
**scATAC-seq**: single-cell assay for transposase-accessible chromatin sequencing
